# SQ-HAF: Source Quality-Guided Hierarchical Adaptation and Multi-Branch Fusion for Few-Shot Cross-Subject SSVEP Recognition

**DOI:** 10.3390/s26185830

**Published:** 2026-09-14

**Authors:** Haoran Lin, Qing He, Yuanbo Zhu, Juanning Si, Fangjun Wang

**Affiliations:** School of Instrumentation Science and Opto-Electronics Engineering, Beijing Information Science and Technology University, Beijing 100192, China; 2024020178@bistu.edu.cn (H.L.); 2024020228@bistu.edu.cn (Y.Z.); sijuanning@bistu.edu.cn (J.S.); 2025020230@bistu.edu.cn (F.W.)

**Keywords:** steady-state visual evoked potential, cross-subject transfer, few-shot calibration, source selection, hierarchical adaptation, covariance alignment, multi-branch fusion

## Abstract

Reducing user-specific calibration is critical for practical steady-state visual evoked potential (SSVEP)-based brain–computer interfaces (BCIs), yet few-shot cross-subject decoding remains challenged by heterogeneous source transferability and inter-subject variability. We propose SQ-HAF, a source quality-guided framework that combines target-relevant source selection, frequency neighborhood-regularized spatial filtering, covariance alignment, and multi-branch decision fusion. Candidate source subjects are ranked primarily by target–source template similarity; when more than one labeled calibration trial per stimulus is available, split-half template consistency (STC) provides a bounded confidence adjustment. The retained source data are used to construct aligned generalized and source-specific templates. For recognition, SQ-HAF fuses a five-subband harmonic reference CCA score with generalized source template and source-specific template scores. We evaluated SQ-HAF using leave-one-subject-out validation on the 35-subject Benchmark and 70-subject BETA datasets. Under the 1.0 s protocol with one labeled calibration trial per stimulus, the complete SQ-HAF configuration achieved 81.57% accuracy (ACC) and 227.83 bits/min information transfer rate (ITR) on Benchmark, and 65.56% ACC and 163.42 bits/min ITR on BETA. In a matched 0.5 s analysis with two calibration trials per stimulus, the five-subband harmonic reference design improved ACC over single-band processing on both datasets after Holm correction. These results indicate that target-relevant source screening and complementary harmonic/template evidence can support low-calibration cross-subject SSVEP decoding.

## 1. Introduction

Brain–computer interfaces (BCIs) establish a direct communication pathway between neural activity and external devices, and noninvasive electroencephalography (EEG) remains one of the most practical modalities for real-world deployment [1,2,3]. Among EEG paradigms, steady-state visual evoked potentials (SSVEPs) are particularly attractive owing to their high information transfer rate, favorable signal-to-noise ratio, and suitability for dense multi-target interaction [4,5,6,7]. Public benchmark resources, including the Benchmark dataset and the BETA database, provide multi-subject, multi-target testbeds for evaluating SSVEP stimulus coding and frequency recognition methods [8,9], while recent studies have broadened practical application scenarios and investigated new feature-modeling strategies [10,11].

The intended application of this work is non-invasive EEG-based brain–computer interfacing. SSVEP-based interfaces can translate visually evoked neural responses into control commands and may support assistive communication, hands-free interaction, and rehabilitation-oriented human–computer interaction [1,3,10]. However, lengthy subject-specific calibration increases preparation time and user burden, which may be particularly undesirable in repeated or assistive-use scenarios [12,13]. Reducing the calibration requirement while preserving reliable cross-subject decoding is therefore relevant to the practical deployment of biomedical EEG interfaces.

Considerable progress has been achieved in SSVEP decoding. Standard harmonic reference-based canonical correlation analysis (CCA) enables calibration-free recognition by comparing EEG responses with sinusoidal reference signals [14,15,16,17]. When calibration data are available, subject-specific templates and task-related component analysis (TRCA)-based spatial filtering can further improve performance by exploiting individual response patterns and cross-trial reproducibility [5,15]. More recent advances, including periodically repeated component analysis, spectrum-enhanced spatial filtering, and dedicated preprocessing strategies, further underscore the importance of learning stable and discriminative SSVEP representations [18,19,20]. Deep learning EEG decoders have also explored convolutional feature extraction combined with Transformer self-attention; EEG Conformer is a representative convolutional–Transformer architecture evaluated on motor imagery and emotion recognition EEG datasets [21]. However, high decoding accuracy still often relies on subject-specific calibration, which increases preparation time and user burden and thus limits practical usability [5,22].

To reduce calibration effort, transfer learning has become a major direction in SSVEP-based BCIs. Cross-subject spatial-filter transfer has been used to reuse an existing user model without collecting training data from a new user, while an improved version addressed trial-to-trial differences in the amplitude and pattern of brain signals [12,23]. Within-subject transfer has also been studied across days and electrode types, with zero-training transfer reported for long-term SSVEP use [13]. Other work transferred template and spatial-filter information across sessions, subjects, and EEG montages using least-squares transformation, and cross-device work aligned spatial patterns and covariance for wet-to-dry electrode transfer [24,25]. Representative-based cold-start source recommendation has also been investigated for adaptive SSVEP-BCI [26]. More general EEG transfer methods align trials in Euclidean space or covariance representations in Riemannian/tangent space so that recordings from different subjects or sessions become more comparable [27,28,29]. Related transfer strategies have used Riemannian similarities between SSVEP users, dimensionality-transcending representations, and semi-supervised calibration to facilitate reuse across heterogeneous users or datasets [30,31,32].

The recent SSVEP literature shows progress along three connected axes. First, source relevance has shifted from using all available auxiliary data toward explicitly identifying useful sources: a 2023 CCA-transfer study used accuracy-based subject selection, whereas later studies used target–source similarity, subject transferability estimation, representative source recommendation, subject correlation, or source–target distance to screen or weight candidate sources [26,33,34,35,36,37]. Second, the transfer representation has expanded from spatial filters and templates to correlation features, aligned signal data, and latent representations. For example, domain generalization methods transfer source-learned filters and templates without target EEG, whereas multi-source variational adaptation jointly learns from labeled source data and unlabeled target data in a latent feature space [22,37,38,39]. Cross-subject assistance, time-weighted fusion, and transfer superposition methods have also been proposed to exploit complementary information across subjects or transfer branches [40,41,42]. Third, the required target calibration has been progressively reduced: published protocols include limited target training data, one trial per stimulus, one class of target calibration data, and a 2 s single-trial calibration segment [22,43,44,45,46]. Neighboring-stimulus data, cross-stimulus transfer, and channel correlation-based domain diversity enhancement have additionally been investigated as ways of exploiting information across stimulus classes or domains [47,48,49,50].

Despite this progress, source selection, alignment, and target calibration are usually designed as separate parts of the transfer pipeline. Existing source selection methods rely on different relevance measures, while alignment may operate on raw trials, covariance matrices, transformed source data, or latent representations [25,27,33,34,35,36,37,39]. The amount and type of target information also vary substantially across studies, ranging from no target EEG or unlabeled target data to one trial per stimulus or other small calibration sets [37,38,43,45,46]. These differences motivate a framework in which source relevance, within-subject spatial filtering, inter-subject covariance alignment, and decision fusion are defined within the same low-calibration protocol.

The scientific contributions of this work are summarized as follows:

- Target-relevant source selection under few-shot calibration. Source subjects are ranked using target–source template similarity, with STC entering as a bounded confidence factor when the calibration budget is large enough to activate the gate. With one calibration trial per stimulus, the rule reduces exactly to template-similarity ranking.
- Frequency neighborhood-regularized spatial filtering and covariance alignment. For each stimulus, Stage 1 estimates a TRCA-style spatial filter by pooling within-class statistics from the central stimulus class and its physically neighboring stimulation frequencies using distance-decayed weights. Stage 2 separately aligns the covariance structure of selected source templates and, when calibration data are available, the target data.
- Five-subband harmonic and template-based decision fusion. The harmonic reference branch combines CCA scores from five fixed subbands, while the other two branches compare the target trial with a generalized source template and weighted source-specific templates. Signed-square fusion combines these three decision cues.

The remainder of this paper is organized as follows. Section 2 introduces the preliminaries, including harmonic reference-based decoding, subject-specific templates and template-based matching, TRCA, and alignment-based transfer. Section 3 presents the proposed source quality evaluation, frequency neighborhood-regularized spatial filtering, covariance alignment, and multi-branch fusion framework. The subsequent sections report the experimental results and analysis.

## 2. Preliminaries

This section briefly reviews the components used in the proposed framework: harmonic reference CCA, subject-specific templates, TRCA, and Euclidean alignment (EA).

For subject *m*, stimulus frequency $f_{k}$, and trial *n*, the EEG trial is denoted by

(1) $$X_{m,k}^{\left(n\right)}\in R^{C\times T},$$

where *C* and *T* are the numbers of channels and time samples, respectively. The corresponding subject-specific template is

(2) $$T_{m,k}=\frac{1}{N_{m}\left(k\right)}\sum_{n=1}^{N_{m}\left(k\right)}X_{m,k}^{\left(n\right)}\in R^{C\times T}.$$

Here, Corr(A,B) denotes the Pearson correlation between the vectorized forms of two matrices, and corr(x,y) denotes the Pearson correlation between two vectors.

### 2.1. Harmonic Reference and Canonical Correlation Analysis

For stimulus frequency $f_{k}$, the harmonic reference is constructed as

(3) $$Y_{k}=\left[\begin{array}{c} \sin(2\pi f_{k}\tau )\\ \cos(2\pi f_{k}\tau )\\ \vdots \\ \sin(2\pi Hf_{k}\tau )\\ \cos(2\pi Hf_{k}\tau ) \end{array}\right]\in R^{2H\times T},$$

where *H* is the number of harmonics and $\tau =[\tau_{1},\ldots ,\tau_{T}]$ is the time index vector. Following standard SSVEP CCA [14], CCA measures the maximal correlation between an EEG trial and $Y_{k}$ by

(4) $$\rho_{k}=\max_{a_{k},b_{k}}\mathrm{corr}\;\left(a_{k}^{⊤}X,b_{k}^{⊤}Y_{k}\right).$$

In standard CCA, the frequency with the largest $\rho_{k}$ is selected. In SQ-HAF, the same harmonic references are evaluated within five weighted EEG subbands and the resulting CCA scores form the harmonic reference branch of the final fusion; the subband construction is given in Section 3.

### 2.2. Subject-Specific Templates and Template-Based Matching

Several SSVEP methods construct templates by averaging or superposing repeated trials, and template-based CCA methods use these templates for recognition [5,15,23]. Therefore, the correlation between subject-specific templates provides a compact measure of structural similarity between SSVEP responses. Here, template correlation is used only as a similarity cue for source evaluation rather than as the final classifier.

### 2.3. Task-Related Component Analysis

TRCA extracts spatial filters that maximize trial reproducibility for the same stimulus [5]. For stimulus $f_{k}$, its basic objective is

(5) $$w_{k}=\arg\max_{w}\frac{w^{⊤}S_{k}^{\mathrm{trca}}w}{w^{⊤}Q_{k}^{\mathrm{trca}}w},$$

where $S_{k}^{\mathrm{trca}}$ is the inter-trial covariance and $Q_{k}^{\mathrm{trca}}$ is the total covariance. Standard TRCA estimates the spatial filter of each stimulus class independently. Stage 1 extends this objective by pooling class-specific TRCA statistics over a small neighborhood defined by physical frequency order, without forming cross-class trial pair covariance terms.

### 2.4. Euclidean Alignment

Cross-subject EEG decoding is hindered by subject-dependent covariance differences. Euclidean alignment (EA) reduces such discrepancies by whitening subject-specific data and recoloring it with a reference covariance [27]:

(6) $$\tilde{X}=C_{\mathrm{ref}}^{1/2}C_{m}^{-1/2}X.$$

This transformation aligns second-order statistics across subjects and forms the basis of Stage 2. In SQ-HAF, the reference covariance is estimated from the selected source subjects, while the target covariance is estimated from labeled calibration trials when they are available.

## 3. Method

### 3.1. Problem Setting and Design Rationale

In a typical SSVEP-based BCI, the user attends to one of several visual stimuli flickering at different frequencies. The visual cortex produces frequency-locked EEG responses, and these responses are recorded using scalp electrodes. The recognition system then determines which stimulus the user attended to and converts this decision into an external control command.

We denote a previous user with labeled EEG as a source subject and the new user as the target subject. The objective is to reuse source subject information while limiting negative transfer and requiring at most a small labeled calibration set from the target subject.

The main difficulty is that SSVEP signal distributions differ among subjects, while the amplitude and pattern of brain signals can also differ across trials; non-stationarity is particularly relevant when short-time signals are used for decoding [23,35,51]. Consequently, transferred templates or models can be useful for a new participant but may also produce negative transfer when source and target responses are poorly matched [35,37]. SQ-HAF addresses these factors with complementary operations: source quality evaluation determines which previous users contribute to the model; Stage 1 estimates stimulus-specific spatial filters within each source subject; Stage 2 aligns second-order covariance across the selected subjects and, when labeled target calibration is available, the target subject; and the final decision combines harmonic reference and template-based evidence.

Stage 1 and Stage 2 operate on different quantities. Stage 1 learns source subject spatial filters from within-subject repeated responses and their local frequency neighborhood. Stage 2 operates in channel space and transforms source templates according to subject-level covariance. The Stage 1 filters are subsequently used in the source-specific template branch, whereas Stage 2 supplies the aligned templates used by the generalized source template branch and the aligned target signal used at prediction time.

### 3.2. Overview

**Figure 1:**
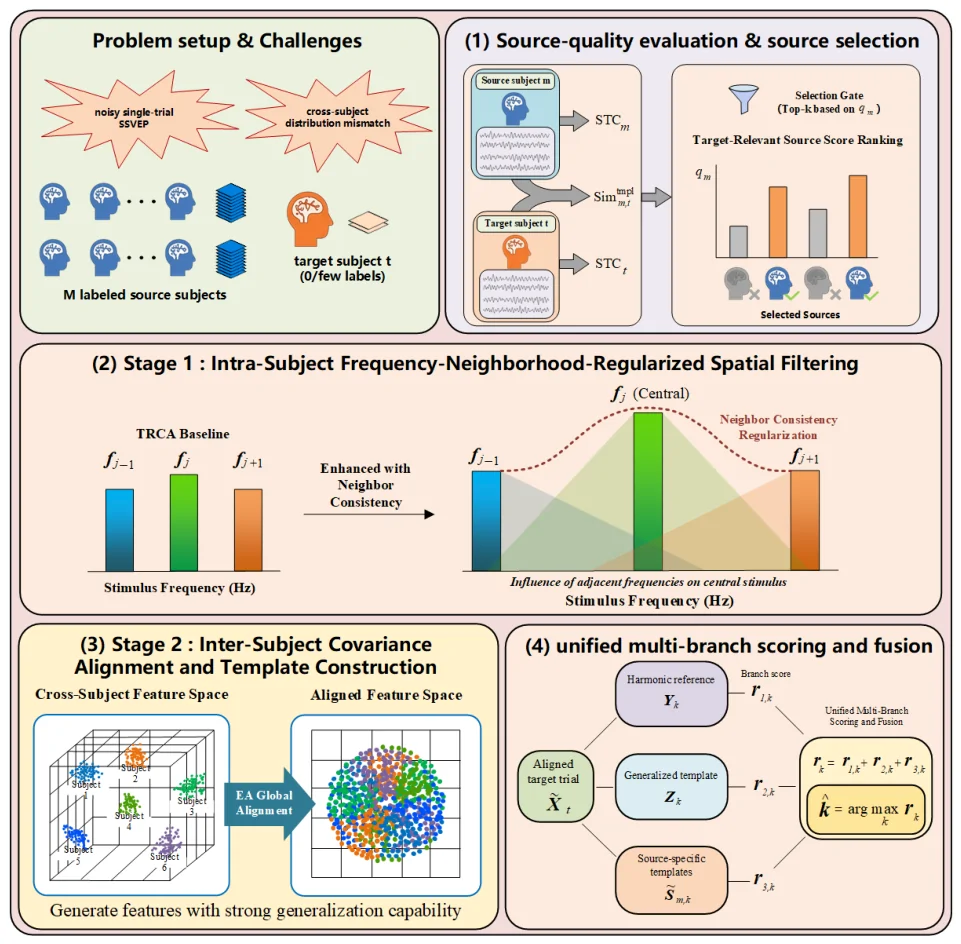
Overview of SQ-HAF for few-shot cross-subject SSVEP recognition. Candidate source subjects are evaluated using target–source template similarity and split-half template consistency (STC) of each source subject, while Stage 1 learns stimulus-specific spatial filters using each target class and its physical frequency neighbors. The highest-ranked source subjects are retained, and Stage 2 aligns their templates in a common covariance space and applies calibration-only target alignment when labeled target trials are available. Recognition combines a five-subband harmonic reference score, a generalized source template score, and a source-specific template score.

We consider *M* labeled source subjects and one target subject *t* with either no labeled calibration data or only a few labeled trials. As illustrated in Figure 1, SQ-HAF contains four functional components:

(1) source quality evaluation and source selection;
(2) Stage 1: intra-subject frequency neighborhood-regularized spatial filtering;
(3) Stage 2: inter-subject covariance alignment and template construction;
(4) multi-branch scoring and fusion.

During fitting, source templates, STC values, and Stage 1 spatial filters are computed for the candidate subjects. Source quality ranking then determines the retained pool and its subject weights; only the retained source models enter Stage 2 and the final template branches. Stage 1 supplies the source-specific spatial filters, whereas Stage 2 supplies covariance-aligned source and target representations and the generalized source template. The harmonic reference branch uses a dedicated five-subband decomposition of the aligned target signal. The three branch scores are combined only at the decision stage.

### 3.3. Source Quality Evaluation and Source Selection

Source–target similarity and split-half template consistency (STC) provide two complementary indicators of potential transfer quality. We therefore characterize candidate source subjects using inter-subject template similarity and STC.

Using the template definition in Equation (2), let $T_{m,k}$ denote the source subject template and let $T_{t,k}$ denote the corresponding template formed from the labeled target-calibration trials. When target calibration is available, source–target template similarity is

(7) $${\mathrm{Sim}}_{m,t}^{\mathrm{tmpl}}=\frac{1}{N_{f}}\sum_{k=1}^{N_{f}}\frac{1+\mathrm{Corr}\;\left(T_{m,k},\;T_{t,k}\right)}{2}.$$

Here, Corr is the vectorized Pearson correlation defined in Section 2. The affine mapping places each class-wise correlation in [0,1] before averaging. When $n_{\mathrm{calib}}=0$, $T_{t,k}$ is unavailable and this similarity is not evaluated.

To quantify intrinsic template reliability, we use split-half template consistency (STC). For the time-ordered estimate used in the main configuration, the trials of source subject *m* and stimulus $f_{k}$ are kept in acquisition order and divided into early and late halves, denoted by A and B. Their templates are

(8) $$T_{m,k}^{\left(A\right)}=\frac{1}{\left|A\right|}\sum_{n\in A}X_{m,k}^{\left(n\right)},\;T_{m,k}^{\left(B\right)}=\frac{1}{\left|B\right|}\sum_{n\in B}X_{m,k}^{\left(n\right)}.$$

Class-wise consistency is measured by the first canonical correlation,

(9) $${\mathrm{STC}}_{m,k}=\rho_{\mathrm{CCA}}\;\left(T_{m,k}^{\left(A\right)},T_{m,k}^{\left(B\right)}\right),$$

and the subject-level score is

(10) $${\mathrm{STC}}_{m}=\frac{1}{N_{f}}\sum_{k=1}^{N_{f}}{\mathrm{STC}}_{m,k}.$$

The time-ordered split measures agreement between early and late templates and therefore can reflect both repeatability and slow temporal changes during acquisition. For comparison, STC can also be formed by random within-class partitioning or by a block-stratified partition based on the observed session/run labels. The block-stratified analysis uses the recorded block information directly rather than inferring blocks from trial indices.

The influence of STC on source ranking depends on the target calibration budget. With one labeled calibration trial per stimulus, the confidence gate defined below is zero, so source ranking depends on target–source template similarity alone. With additional calibration trials, the gate activates the bounded STC correction.

The source quality score is adapted to the amount of labeled target calibration data. Without target calibration, source quality is determined by ${\mathrm{STC}}_{m}$. Once target calibration is available, target–source template similarity provides the transferability term, and STC modulates this term through a bounded confidence factor controlled by $g(n_{\mathrm{calib}})$. Let λ∈[0,1] denote the confidence coefficient; λ=0.2 is used in the experiments. The source quality score is

(11) $$q_{m}=\left\{\begin{array}{cc} {\mathrm{STC}}_{m},\; & n_{\mathrm{calib}}=0,\\ {\mathrm{Sim}}_{m,t}^{\mathrm{tmpl}}\left[1-\lambda \;g\;\left(n_{\mathrm{calib}}\right)\left(1-{\mathrm{STC}}_{m}\right)\right],\; & n_{\mathrm{calib}}\geq 1, \end{array}\right.$$

The confidence gate is defined as

(12) $$g\;\left(n_{\mathrm{calib}}\right)=\mathrm{clip}\left(\frac{n_{\mathrm{calib}}-1}{2},\;0,\;1\right).$$

For one calibration trial per stimulus, this definition gives

(13) $$g\left(1\right)=0,\;q_{m}={\mathrm{Sim}}_{m,t}^{\mathrm{tmpl}}.$$

For $n_{\mathrm{calib}}=1$, the bracketed confidence factor in Equation (11) equals one and the ranking is determined entirely by template similarity. For larger calibration budgets, lower STC reduces the score by at most a fraction controlled by λ, so target relevance remains the leading term.

All candidate source subjects are ranked according to $q_{m}$, and the top $M_{s}$ subjects form the retained source pool $M_{s}$. Their normalized weights are defined as

(14) $$\beta_{m}=\frac{q_{m}}{\sum_{i\in M_{s}}q_{i}},\;m\in M_{s},$$

such that $\sum_{m\in M_{s}}\beta_{m}=1$. This calibration-dependent formulation provides a simple mechanism for incorporating transferability and reliability, and their relative contributions are further examined through ablation analysis.

### 3.4. Stage 1: Intra-Subject Frequency Neighborhood-Regularized Spatial Filtering

Stage 1 estimates a stimulus-specific spatial filter for each source subject. The construction follows a TRCA-style generalized eigenvalue problem and regularizes the class-specific estimate by pooling within-class statistics from a local neighborhood defined by the physical order of the stimulation frequencies. The pooling is performed at the level of class-wise covariance statistics; trials from different stimulus classes are not paired with one another. Let $K=\{1,\ldots ,N_{f}\}$ denote the stimulus-class index set, and let ${\mathrm{rank}}_{f}\left(k\right)$ be the position of class *k* after sorting the stimulation frequencies in ascending order. For neighborhood radius *r*,

(15) $$N_{r}\left(k\right)=\left\{\ell \in K:\left|{\mathrm{rank}}_{f}\left(\ell \right)-{\mathrm{rank}}_{f}\left(k\right)\right|\leq r\right\}.$$

The normalized physical frequency distance is

(16) $$d_{k\ell }=\frac{|f_{k}-f_{\ell }|}{\Delta_{f}},\;\Delta_{f}=\mathrm{median}\left\{f_{\left(j+1\right)}-f_{\left(j\right)}:f_{\left(j+1\right)}>f_{\left(j\right)}\right\},$$

and the class pair weight is

(17) $$\omega_{k\ell }=\left\{\begin{array}{cc} 1, & \ell =k,\\ \gamma \exp(-\eta d_{k\ell }), & \ell \neq k, \end{array}\right.$$

where γ controls the contribution of neighboring classes and η controls distance decay. We use r=1, γ=1, and η=0.5. For joint frequency–phase modulation (JFPM) stimulus coding, the neighborhood is determined only by the ordered stimulation frequencies; phase labels are not merged or averaged.

For source subject *m*, let ${\bar{X}}_{m,\ell }^{\left(i\right)}$ denote trial *i* after removing the temporal mean of each channel. The within-class statistics are

(18) $$Q_{m,\ell }=\sum_{i}{\bar{X}}_{m,\ell }^{\left(i\right)}{\bar{X}}_{m,\ell }^{(i)⊤},\;G_{m,\ell }=\sum_{i<j}\left({\bar{X}}_{m,\ell }^{\left(i\right)}{\bar{X}}_{m,\ell }^{(j)⊤}+{\bar{X}}_{m,\ell }^{\left(j\right)}{\bar{X}}_{m,\ell }^{(i)⊤}\right).$$

The local statistics for class *k* are

(19) $$G_{m,k}^{\mathrm{loc}}=\sum_{\ell \in N_{r}\left(k\right)}\omega_{k\ell }G_{m,\ell },\;Q_{m,k}^{\mathrm{loc}}=\sum_{\ell \in N_{r}\left(k\right)}\omega_{k\ell }Q_{m,\ell }.$$

The Stage 1 filter is the leading generalized eigenvector,

(20) $$w_{m,k}=\arg\max_{w\neq 0}\frac{w^{⊤}G_{m,k}^{\mathrm{loc}}w}{w^{⊤}\left(Q_{m,k}^{\mathrm{loc}}+\delta \;\frac{\mathrm{tr}(Q_{m,k}^{\mathrm{loc}})}{C}I\right)w},$$

where $\delta =10^{-6}$ is the trace-scaled numerical regularization coefficient. Equivalently, $w_{m,k}$ is the dominant generalized eigenvector of the matrix pair in Equation (20).

Stage 1 estimates the stimulus-specific spatial filters $w_{m,k}$ used by the source-specific template branch. Stage 2, described next, independently estimates subject-level covariance from the channel-space trials and aligns the source templates.

### 3.5. Stage 2: Inter-Subject Covariance Alignment and Template Construction

Stage 2 reduces subject-dependent covariance differences in channel space. For a temporally centered trial $\bar{X}\in R^{C\times T}$, define the trace regularization operator

(21) $$R_{\delta }\left(C\right)=\frac{C+C^{⊤}}{2}+\delta \frac{\mathrm{tr}(C)}{C}I,$$

and the regularized trial covariance

(22) $${\mathrm{Cov}}_{\delta }\left(X\right)=R_{\delta }\left(\frac{\bar{X}{\bar{X}}^{⊤}}{\max(T-1,1)}\right).$$

For retained source subject *m*, the subject covariance is

(23) $$C_{m}=R_{\delta }\left(\frac{1}{N_{m}^{\mathrm{all}}}\sum_{k=1}^{N_{f}}\sum_{n=1}^{N_{m}\left(k\right)}{\mathrm{Cov}}_{\delta }\left(X_{m,k}^{\left(n\right)}\right)\right),\;N_{m}^{\mathrm{all}}=\sum_{k=1}^{N_{f}}N_{m}\left(k\right).$$

The reference covariance is the arithmetic mean over the selected source subjects,

(24) $$C_{\mathrm{ref}}=\frac{1}{M_{s}}\sum_{m\in M_{s}}C_{m}.$$

The source transform and aligned source template are

(25) $$A_{m}=C_{\mathrm{ref}}^{1/2}C_{m}^{-1/2},\;{\tilde{T}}_{m,k}=A_{m}T_{m,k}.$$

When labeled target calibration trials are available, the target covariance is estimated from those trials only:

(26) $$C_{t}^{\mathrm{cal}}=R_{\delta }\left(\frac{1}{|C_{t}|}\sum_{X\in C_{t}}{\mathrm{Cov}}_{\delta }\left(X\right)\right),\;A_{t}=C_{\mathrm{ref}}^{1/2}{\left(C_{t}^{\mathrm{cal}}\right)}^{-1/2}.$$

A test trial is then transformed as ${\tilde{X}}_{t}=A_{t}X_{t}$. Held-out target test trials are not used to estimate $C_{t}^{\mathrm{cal}}$. When no labeled target calibration is available, the target-side covariance transform is not applied.

The generalized source template is formed from the aligned templates using the source weights:

(27) $$Z_{k}=\sum_{m\in M_{s}}\beta_{m}{\tilde{T}}_{m,k}.$$

For the generalized source template branch, a class-specific cross-subject spatial filter $v_{k}$ is also learned. Let

(28) $$R_{k}=\sum_{m\in M_{s}}\beta_{m}{\mathrm{Cov}}_{\delta }\left({\tilde{T}}_{m,k}\right),$$

and

(29) $$P_{k}=\sum_{\begin{array}{c} i,j\in M_{s}\\ i\neq j \end{array}}\beta_{i}\beta_{j}\frac{{\bar{\tilde{T}}}_{i,k}{\bar{\tilde{T}}}_{j,k}^{⊤}}{\max(T-1,1)},$$

where ${\bar{\tilde{T}}}_{m,k}$ denotes the temporally centered aligned source template. The filter $v_{k}$ is the leading generalized eigenvector of $\left(P_{k},R_{k}\right)$ with the same trace-scaled diagonal regularization used for the generalized eigenvalue solver.

### 3.6. Unified Multi-Branch Scoring and Fusion

Given the aligned target trial ${\tilde{X}}_{t}$, SQ-HAF evaluates each candidate stimulus $f_{k}$ using three branches. The harmonic reference branch uses a dedicated five-subband filter bank that is separate from the channel-space processing used to construct the source templates. For subband h∈{1,…,5}, a third-order Butterworth band-pass filter, applied with zero-phase forward–backward filtering, is defined by

(30) $$F_{h}:\;\left[6h,\;90\right]\;\mathrm{Hz},\;h=1,\ldots ,5,$$

corresponding to the passbands [6,90], [12,90], [18,90], [24,90], and [30,90] Hz. The subband weights are

(31) $$\alpha_{h}=\frac{h^{-1.25}+0.25}{\sum_{j=1}^{5}\left(j^{-1.25}+0.25\right)},\;\sum_{h=1}^{5}\alpha_{h}=1.$$

For candidate *k*, the CCA score in subband *h* is

(32) $$\rho_{h,k}^{\mathrm{cca}}=\max_{a_{h,k},b_{h,k}}\mathrm{corr}\left(a_{h,k}^{⊤}F_{h}\left({\tilde{X}}_{t}\right),b_{h,k}^{⊤}Y_{k}\right),$$

and the harmonic reference score before nonlinear fusion is

(33) $$u_{1,k}=\sum_{h=1}^{5}\alpha_{h}\rho_{h,k}^{\mathrm{cca}}.$$

The generalized source template branch compares the target trial and generalized template after projection by the cross-subject filter:

(34) $$u_{2,k}=\mathrm{corr}\left(v_{k}^{⊤}{\tilde{X}}_{t},v_{k}^{⊤}Z_{k}\right).$$

The source-specific branch uses the Stage 1 filters and source weights:

(35) $$u_{3,k}=\sum_{m\in M_{s}}\beta_{m}\;\mathrm{corr}\left(w_{m,k}^{⊤}{\tilde{X}}_{t},w_{m,k}^{⊤}{\tilde{T}}_{m,k}\right).$$

The three branch scores are transformed by

(36) $$\Phi \left(u\right)=\mathrm{sign}\left(u\right)u^{2}$$

and fused as

(37) $$r_{k}=\Phi \left(u_{1,k}\right)+\Phi \left(u_{2,k}\right)+\Phi \left(u_{3,k}\right).$$

The predicted stimulus is

(38) $$\hat{k}=\arg\max_{k\in \{1,\ldots ,N_{f}\}}r_{k},\;f_{\mathrm{target}}=f_{\hat{k}}.$$

The harmonic reference branch contributes analytical frequency evidence, the generalized source template branch represents structure shared across the selected source subjects, and the source-specific template branch retains subject-dependent template information.

## 4. Experimental Results and Analysis

### 4.1. Datasets and Data Preprocessing

We evaluated the proposed method on two public 40-target SSVEP datasets, namely the Benchmark dataset and the BETA dataset. The Benchmark dataset contains EEG recordings from 35 subjects, while the BETA dataset contains EEG recordings from 70 subjects. Both datasets have been widely used for evaluating SSVEP recognition and cross-subject transfer learning methods. To ensure a comparable evaluation protocol across datasets, the same channel configuration, frequency band, visual latency setting, EEG window lengths, and recognition metrics were adopted whenever possible.

#### 4.1.1. Dataset 1: Benchmark Dataset

For the Benchmark dataset [8], a visual latency of 0.14 s was considered during EEG data segmentation. EEG data from [0.64,2.64] s were extracted to obtain a 2 s steady-state SSVEP segment, where the starting time accounts for the experimental cuing period and the 0.14 s visual latency. The data were band-pass filtered within [5,90] Hz. Nine occipitoparietal channels were selected, including Pz, PO5, PO3, POz, PO4, PO6, O1, Oz, and O2.

#### 4.1.2. Dataset 2: BETA Dataset

For the BETA dataset [9], the same 0.14 s visual latency was adopted to maintain a unified preprocessing protocol. Under the 3 s stimulus condition, EEG data from [0.64,2.64] s were extracted, yielding a 2 s SSVEP segment consistent with the Benchmark dataset. Rather than using the officially preprocessed version, we used the raw, unfiltered WOF version of the BETA dataset. Therefore, the same [5,90] Hz band-pass filtering procedure was applied. The same nine occipitoparietal channels, namely Pz, PO5, PO3, POz, PO4, PO6, O1, Oz, and O2, were selected.

### 4.2. Experimental Protocol and Parameter Settings

The proposed method, denoted as SQ-HAF, was compared with the SSVEP unsupervised transfer learning (SUTL) framework [35], the inter- and intra-subject transfer learning framework (IISTLF) [45], ensemble task-related component analysis (eTRCA) [5], transfer template-based canonical correlation analysis (TtCCA) [52], task-discriminant component analysis (TDCA) [53], and a supervised EEG-Conformer baseline [21]. These methods cover representative calibration-based, cross-subject, transfer-based, and deep learning SSVEP recognition strategies under the evaluated conditions.

All experiments were conducted using a leave-one-subject-out (LOSO) validation protocol. In each fold, one subject was selected as the target subject, while the remaining subjects were used as candidate source subjects. The target subject was strictly excluded from the source pool. Source subject selection, source quality estimation, alignment, and model construction were performed using only source subject data and the labeled calibration trials of the target subject. The target subject test trials were never used during source selection or model training, thereby avoiding information leakage. The implementations of several comparison methods were developed or adapted with the support of the MetaBCI platform [54]. The LOSO target/source partition and calibration/test separation were matched where permitted by each implementation, although preprocessing and calibration conventions differed for some comparators.

The LOSO setting has a direct user generalization interpretation: in each fold, the held-out subject represents a new user whose test trials are unavailable during model construction. This design therefore evaluates whether information learned from previous users can support recognition for a new user with limited or no calibration data.

Unless otherwise specified, SQ-HAF was evaluated with $n_{\mathrm{calib}}=1$, where $n_{\mathrm{calib}}$ denotes the number of labeled calibration trials per stimulus frequency for the target subject. SQ-HAF used the Stage 1 physical frequency neighborhood, time-ordered STC, the calibration-dependent source quality score in Equation (11), calibration-only target alignment, and the five-subband harmonic reference branch defined in Section 3. Calibration and test trials were disjoint in every fold. For the source-transfer comparison, SQ-HAF and SUTL used the same source pool size. When repeated calibration splits were used, results were first averaged within each target subject and then summarized across target subjects under LOSO validation.

Unless otherwise specified, the number of harmonics was set to H=3. SQ-HAF used five harmonic subbands with lower cutoffs of 6, 12, 18, 24, and 30 Hz and a common upper cutoff of 90 Hz; the normalized subband weights follow Equation (31). The key parameter settings are summarized in Table 1. In the IISTLF runs, one calibration trial was sampled without replacement for each stimulus class, matching the $n_{\mathrm{calib}}=1$ convention used for the SQ-HAF comparison. Calibration trials were excluded from the test pool in each LOSO fold.

### 4.3. Computational Complexity Analysis

Let *M* be the number of candidate source subjects, $M_{s}$ the number of selected source subjects, $N_{f}$ the number of stimulus classes, *L* the mean number of source trials per class, *C* the number of EEG channels, and *T* the number of samples per trial. Let *R* denote the maximum Stage 1 neighborhood size and $B_{h}$ the number of harmonic reference subbands; in the reported configuration, R≤3 and $B_{h}=5$.

Keeping only the dominant data-dependent terms, model fitting scales approximately as

(39) $$O\;\left(N_{f}C^{2}T\left(MRL^{2}+M_{s}^{2}\right)\right),$$

which covers the Stage 1 covariance statistics and the cross-subject template construction. For $N_{\mathrm{test}}$ target trials, the dominant prediction cost is

(40) $$O\;\left(N_{\mathrm{test}}N_{f}CT\left(B_{h}C+M_{s}\right)\right),$$

where the $B_{h}$ term arises from harmonic reference CCA and the $M_{s}$ term from source template matching. Thus, the five-subband design increases only the harmonic reference part of prediction linearly with $B_{h}$; Stage 1 and Stage 2 fitting are not repeated across the harmonic subbands. Storage grows linearly with the retained source templates and the target representations used for prediction.

### 4.4. Evaluation Metrics and Result Aggregation

SQ-HAF was evaluated at EEG window lengths of 0.5, 1.0, 1.5, and 2.0 s. Accuracy (ACC) and information transfer rate (ITR) were used as evaluation metrics. The ITR was computed using the standard BCI communication rate formula:

(41) $$\mathrm{ITR}=\left[\log_{2}N_{f}+P\log_{2}P+\left(1-P\right)\log_{2}\;\left(\frac{1-P}{N_{f}-1}\right)\right]\frac{60}{T_{\mathrm{win}}}$$

where *P* denotes recognition accuracy in probability form, $N_{f}$ is the number of candidate stimulus frequencies, and $T_{\mathrm{win}}$ is the EEG window length used for recognition.

For the window-length analysis, $T_{\mathrm{win}}$ was set to 0.5, 1.0, 1.5, or 2.0 s according to the evaluated condition. The 0.14 s visual latency was considered during data segmentation but was not included in the ITR denominator. Additional gaze shifting or cueing time was also not included. The reported ITR therefore represents an offline window-based communication rate used for comparison under identical EEG data lengths.

LOSO results are summarized across target subjects. For repeated calibration splits, the repeated runs were first averaged within each target subject before the across-subject summary was formed. The 1.0 s component analysis evaluates SQ-HAF and its component variants under $n_{\mathrm{calib}}=0,1,2$; the main text focuses on the one-trial operating point, and the complete three-budget matrices are provided in Appendix A.

For the matched 0.5 s harmonic-processing analysis at $n_{\mathrm{calib}}=2$, identical LOSO folds, target calibration/test partitions, and random seeds were used across the five-subband, single-band, and no-harmonic reference conditions. The statistical unit in this analysis was the matched target–seed record (35 subjects × 5 seeds for Benchmark and 70 subjects × 5 seeds for BETA). Mean ACC differences were accompanied by paired *t*-based 95% confidence intervals, while distributional differences were assessed with the Wilcoxon signed-rank test. Holm correction was applied across the five prespecified ACC comparisons within each dataset. The 1.0, 1.5, and 2.0 s harmonic reference comparisons are presented descriptively.

Stage 1 was additionally examined at 1.0 s and $n_{\mathrm{calib}}=1$ using matched LOSO comparisons. Physical adjacency was defined after sorting the actual stimulation frequencies. Stage 1 and the corresponding no-Stage 1 condition used identical target folds and three random seeds; seed-level results were averaged within each target subject before paired inference. ACC and the subject-level adjacent confusion statistic were assessed with paired Wilcoxon signed-rank tests and paired bootstrap 95% CIs, with Holm correction across the four dataset–metric comparisons. A neighborhood strength analysis evaluated γ∈{0,0.1,0.2,0.4,0.8,1.0} while holding the remaining settings fixed; γ=1.0 is the value used by SQ-HAF. These component-level analyses use paired differences to isolate the contribution of Stage 1.

For STC partition sensitivity, the time-ordered rule was compared with both random within-class partitioning and a block-stratified alternative based on observed session/run labels. The block-stratified analysis covered $n_{\mathrm{calib}}=0,1,2$ on both datasets. Paired bootstrap 95% CIs and Wilcoxon signed-rank tests were computed at the target subject level; Holm correction was applied within each metric across the six dataset–calibration comparisons. At $n_{\mathrm{calib}}=1$, the confidence gate is identically zero, so STC partitioning cannot change the final source quality score in that condition.

### 4.5. Overall Comparison with Existing Methods

Table 2 summarizes recognition performance on the Benchmark and BETA datasets across the 0.5, 1.0, 1.5, and 2.0 s EEG windows. The SQ-HAF entries correspond to the complete configuration with one labeled calibration trial per stimulus, including the five-subband harmonic reference branch defined in Section 3. The comparison includes calibration-based, transfer-based, and deep learning baselines under the settings described above.

**Figure 2:**
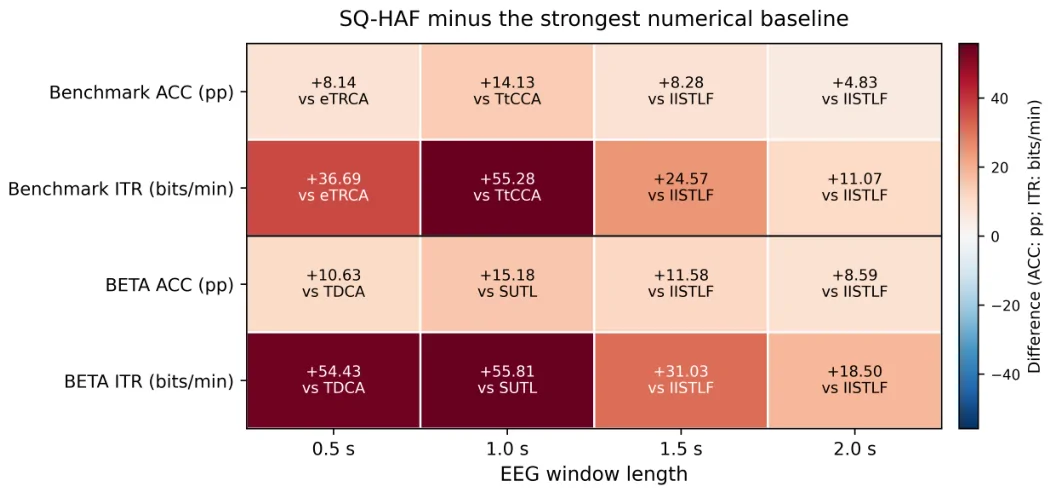
Performance difference between SQ-HAF and the strongest numerical baseline under each dataset–window–metric condition. Cell values are calculated as SQ-HAF minus the strongest baseline.

To complement the aggregate values in Table 2, Figure 2 summarizes the difference between SQ-HAF and the strongest numerical baseline for each dataset–window–metric condition. The strongest baseline is identified separately for ACC and ITR; positive values indicate higher performance for SQ-HAF, whereas negative values indicate higher performance for the corresponding baseline.

At 0.5 s, the complete SQ-HAF configuration achieved 43.81% ACC and 177.08 bits/min on Benchmark and 36.53% ACC and 135.30 bits/min on BETA. At the one-trial 1.0 s operating point, the corresponding results increased to 81.57%/227.83 bits/min and 65.56%/163.42 bits/min, respectively. SQ-HAF obtained the highest ACC and ITR among the listed methods at all four window lengths on both datasets. ACC further increased to 90.57% and 94.96% on Benchmark at 1.5 and 2.0 s, and to 79.34% and 85.53% on BETA.

The supervised EEG-Conformer baseline achieved accuracies of 8.46±3.89%, 35.81±17.63%, 57.39±22.20%, and 67.14±21.42% on Benchmark across the 0.5–2.0 s windows, and 7.45±3.90%, 27.99±14.80%, 34.84±18.35%, and 49.17±22.92% on BETA. These values provide a deep learning reference under the evaluated LOSO setting.

### 4.6. Effect of Window Length and Dataset Characteristics

The four-window results in Table 2 show a consistent accuracy–speed trade-off for SQ-HAF. Mean ACC increased monotonically from 43.81% at 0.5 s to 94.96% at 2.0 s on Benchmark and from 36.53% to 85.53% on BETA. Mean ITR peaked at 1.0 s, reaching 227.83 bits/min on Benchmark and 163.42 bits/min on BETA, and then decreased at longer windows as the additional observation time outweighed the accuracy gain. The 1.0 s window was therefore used for the detailed component analysis.

### 4.7. Effect of Calibration Effort

To examine the influence of target subject calibration effort, SQ-HAF was evaluated at 1.0 s with zero, one, or two labeled calibration trials per stimulus. Table 3 reports the corresponding LOSO summaries.

**Figure 3:**
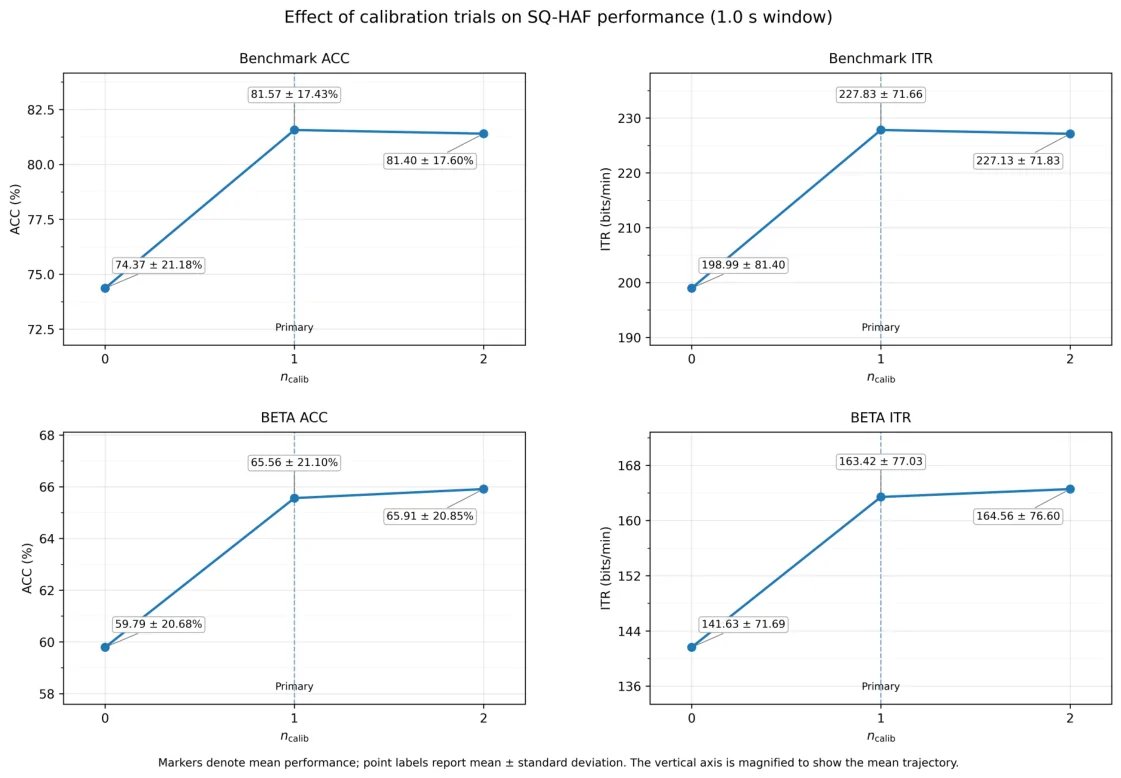
Effect of the number of target calibration trials on SQ-HAF at a 1.0 s EEG window. Panels show ACC and ITR on Benchmark and BETA for $n_{\mathrm{calib}}=0,1,2$. Markers indicate the mean performance and the point annotations report mean ± standard deviation across target subjects.

Figure 3 provides a visual summary of the calibration-effort analysis at the 1.0 s operating window. The mean trajectories show how ACC and ITR change from zero to two labeled calibration trials per stimulus, while the annotations report the corresponding mean and standard deviation for each operating point.

On Benchmark, introducing one labeled calibration trial per stimulus increased ACC from 74.37% to 81.57% and ITR from 198.99 to 227.83 bits/min. On BETA, the corresponding change was from 59.79% to 65.56% ACC and from 141.63 to 163.42 bits/min. Increasing the calibration budget from one to two trials produced only a small change in the aggregate means in both datasets.

With one calibration trial per stimulus, g(1)=0, so Equation (11) reduces to similarity-only source ranking. The improvement from zero to one calibration trial therefore reflects the availability of target-specific template information rather than an STC confidence effect.

### 4.8. Ablation and Component Analysis

The 1.0 s ablation analysis used the complete five-subband configuration as the reference. Each ablation changed one source selection, spatial filtering, covariance alignment, decision-branch, or fusion component while leaving the remaining components unchanged. Table 4 reports the one-trial ACC and ITR results, while Appendix A provides compact ACC matrices across all three calibration budgets for the principal structural ablations.

**Figure 4:**
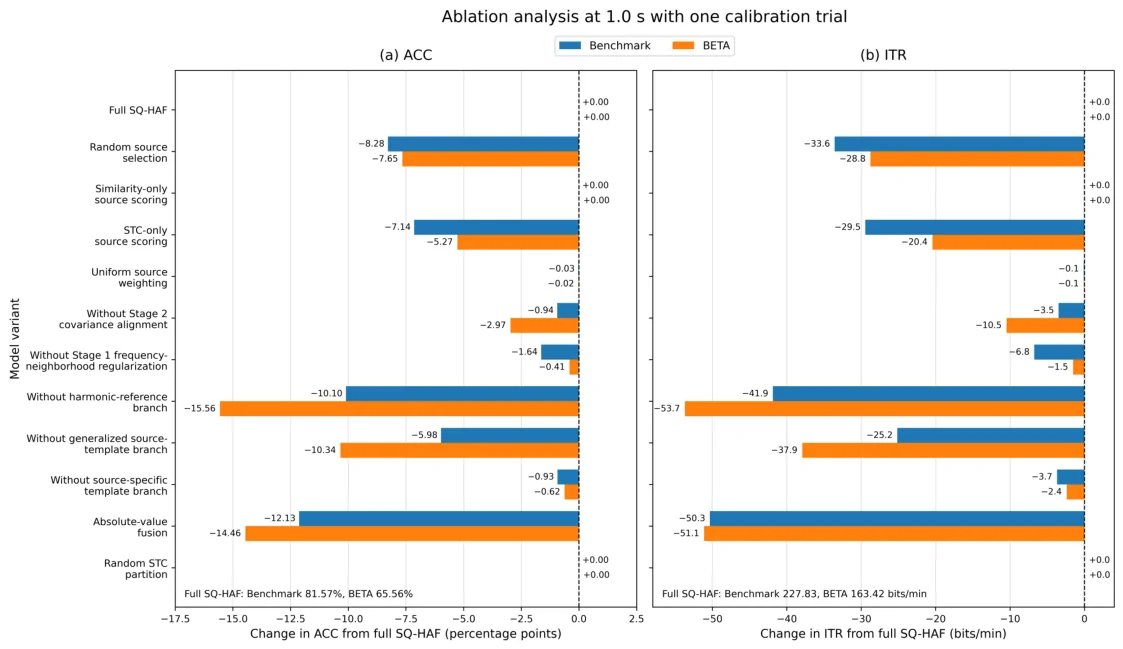
Controlled ablation analysis of SQ-HAF at 1.0 s with one calibration trial per stimulus. The panels show changes in ACC and ITR relative to the complete five-subband SQ-HAF configuration within the same ablation run; negative values indicate performance degradation after the corresponding component change.

Figure 4 visualizes the same controlled one-trial ablation results as changes relative to the complete five-subband SQ-HAF configuration. This representation emphasizes the direction and magnitude of each component’s effect while Table 4 retains the corresponding absolute ACC and ITR values.

Replacing target-relevant source selection with random source selection reduced ACC by 8.28 percentage points on Benchmark and 7.65 percentage points on BETA, with ITR decreases of 33.57 and 28.75 bits/min, respectively. STC-only source scoring was also lower than SQ-HAF by 7.14 and 5.27 percentage points. In contrast, similarity-only source scoring reproduced the SQ-HAF one-trial result exactly, as expected from g(1)=0, and uniform source weighting changed the aggregate means only slightly.

Removing Stage 1 reduced ACC by 1.64 percentage points on Benchmark and 0.41 percentage points on BETA. Removing Stage 2 reduced ACC by 0.94 and 2.97 percentage points, respectively. Among the decision branches, removing the generalized source template branch reduced ACC by 5.98 percentage points on Benchmark and 10.34 percentage points on BETA, whereas removing the source-specific template branch reduced ACC by 0.93 and 0.62 percentage points. Removing the five-subband harmonic reference branch produced larger decreases of 10.10 and 15.56 percentage points, with ITR decreases of 41.86 and 53.69 bits/min. Replacing signed-square fusion with absolute value fusion reduced ACC by 12.13 percentage points on Benchmark and 14.46 percentage points on BETA.

### 4.9. Stage 1 Frequency Neighborhood Analysis

The Stage 1 neighborhood in Equation (15) follows the physical order of the 40 stimulation frequencies and pools class-specific TRCA statistics without averaging temporal or phase responses across classes. Its class pair behavior was evaluated by comparing Stage 1 with the corresponding no-Stage 1 condition at 1.0 s and $n_{\mathrm{calib}}=1$. Table 5 lists representative directed adjacent frequency cells with the largest absolute count changes.

Across all adjacent frequency cells, Benchmark contained 557 adjacent errors with Stage 1 and 657 without Stage 1, corresponding to 2.652% and 3.129% of all evaluated trials, respectively. BETA contained 1268 and 1267 adjacent errors, corresponding to 5.032% and 5.028%. Thus, the aggregate neighboring-class pattern differed across datasets rather than showing a uniform reduction.

The target subject paired analysis is summarized in Table 6. On Benchmark, Stage 1 increased ACC by 1.8524 percentage points on average and reduced the subject-level adjacent confusion statistic by 1.1830 percentage points; both comparisons remained significant after Holm correction. On BETA, the corresponding ACC and adjacent confusion differences were small and did not remain significant after Holm correction.

We also varied the Stage 1 neighborhood strength coefficient γ while keeping the neighborhood radius and decay fixed; λ remains the source quality confidence coefficient in Equation (11). Table 7 reports the ACC change relative to the SQ-HAF setting γ=1.0 and the corresponding Holm-adjusted paired *p* values. Across the tested grid, the largest absolute ACC change was 0.1191 pp on Benchmark and 0.1627 pp on BETA, and none of the comparisons with the default setting was significant after Holm correction. The paired adjacent confusion comparisons were likewise non-significant after correction (all adjusted p≥0.6447).

### 4.10. Effect of STC Partition

The time-ordered and random within-class STC partitions produced closely matched results in the five-subband 1.0 s analysis. With one calibration trial per stimulus, the two partitions were identical because g(1)=0. Under zero calibration, the random partition changed ACC by +0.05 pp on Benchmark and –0.02 pp on BETA relative to the time-ordered split; under two calibration trials, the changes were –0.04 and –0.02 pp, respectively.

A block-stratified alternative based on the observed session/run labels was also evaluated. Table 8 reports paired differences relative to the time-ordered partition. At $n_{\mathrm{calib}}=0$ and 2, the ACC and ITR differences were small on both datasets, and none was significant after Holm correction. At $n_{\mathrm{calib}}=1$, the differences were exactly zero because the confidence gate in Equation (12) is inactive. The source ranking nevertheless changed under block-stratified partitioning in the zero- and two-calibration analyses, showing that the partition changes the STC-derived ordering even when its aggregate recognition effect is small.

Recognition performance was closely matched across the time-ordered, random, and block-stratified STC partitions under the tested calibration budgets. The time-ordered definition is retained as the main STC construction and represents consistency between early and late source templates.

### 4.11. Effect of Harmonic Subband Design

The harmonic reference branch was examined separately through a controlled branch-level comparison of five-subband processing, single-band processing, and removal of harmonic reference scoring. This analysis isolates the effect of harmonic reference processing from the complete SQ-HAF result reported in Table 2. At 0.5 s, Table 9 reports the aggregate mean ACC and ITR for all three calibration budgets.

With one calibration trial per stimulus, the 0.5 s summaries in Table 9 show higher ACC and ITR for the five-subband design than for the single-band design on both datasets. Relative to removing harmonic reference scoring, the ACC gains were 9.01 percentage points on Benchmark and 10.26 percentage points on BETA. The same ordering was observed with zero and two calibration trials. For $n_{\mathrm{calib}}=2$, the matched ACC analysis in Table 10 directly quantifies the five-subband versus single-band difference and the single-band versus no-harmonic reference difference.

Within the complete 1.0 s SQ-HAF ablation in Table 4, removing the harmonic reference branch produced the largest branch-specific decrease on both datasets. In the separate harmonic-processing comparison, the five-subband condition achieved 78.15% ACC/214.39 bits/min on Benchmark, compared with 68.41%/175.65 bits/min for single-band processing and 63.63%/156.33 bits/min without harmonic reference scoring. The corresponding BETA results were 63.22%/153.55 bits/min, 51.12%/113.92 bits/min, and 45.81%/96.22 bits/min.

**Figure 5:**
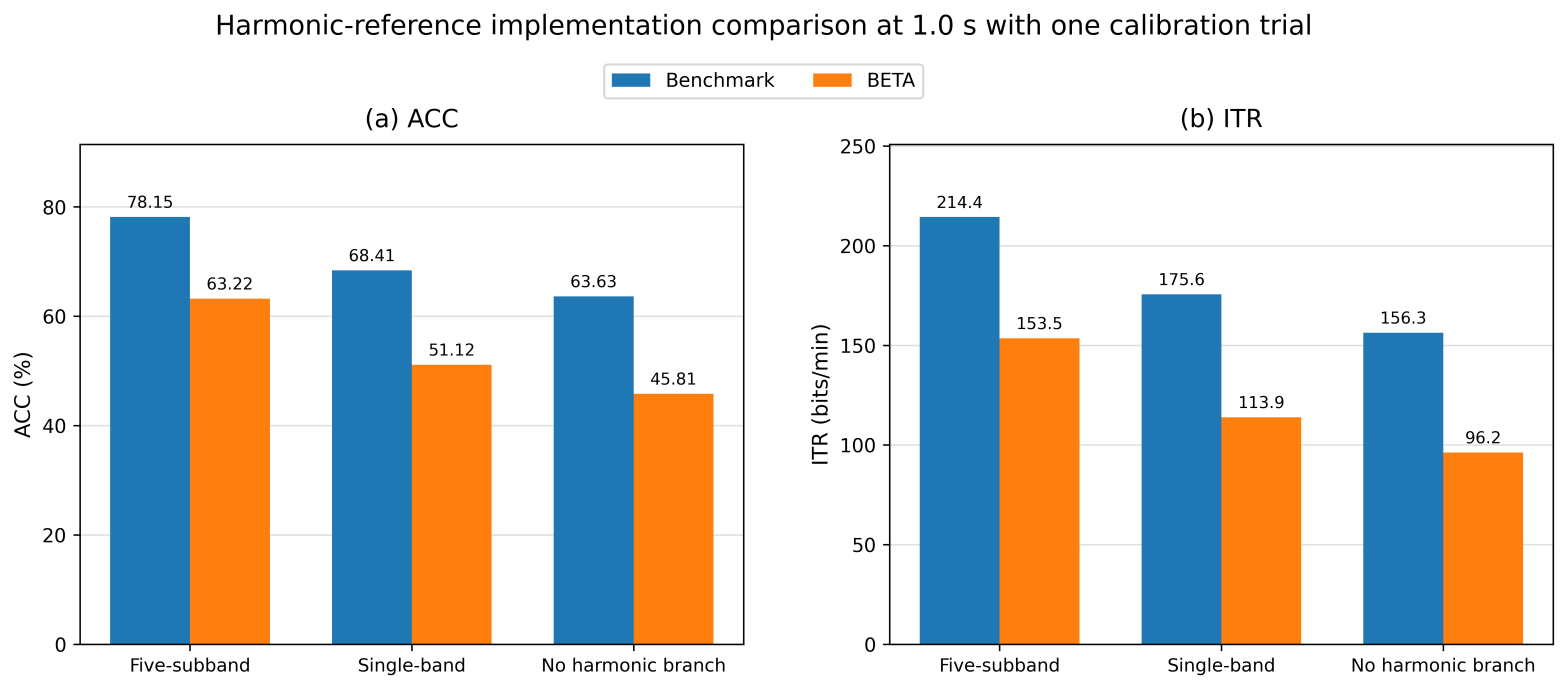
Branch-level comparison of harmonic reference processing at 1.0 s with one calibration trial per stimulus. Five-subband processing is compared with single-band processing and with removal of harmonic reference scoring. Panels report ACC and ITR on Benchmark and BETA.

To distinguish the effect of harmonic subband processing from the contribution of the harmonic reference branch itself, Figure 5 compares the five-subband design with a single-band implementation and with removal of the harmonic reference branch at 1.0 s and $n_{\mathrm{calib}}=1$. The five-subband design achieved the highest ACC and ITR on both datasets, while the single-band implementation remained above the no-harmonic reference condition. This ordering shows that the performance gain is associated not only with retaining harmonic reference evidence, but also with processing that evidence across weighted subbands.

Table 10 reports the matched 0.5 s ACC comparisons at $n_{\mathrm{calib}}=2$. The five-subband design exceeded the single-band design by 7.4857 percentage points on Benchmark and 7.1321 percentage points on BETA. The corresponding Holm-adjusted Wilcoxon *p* values were $8.769\times 10^{-27}$ and $9.201\times 10^{-50}$, respectively. Comparing single-band processing with removal of harmonic reference scoring yielded a smaller 1.5679-percentage-point difference on Benchmark, which was not significant after Holm correction, and a 3.3071-percentage-point difference on BETA, which remained significant after correction.

At 1.5 and 2.0 s, the same three branch-level harmonic reference conditions were compared at the one-trial operating point, as summarized in Table 11. The complete SQ-HAF performance is reported separately in Table 2. The matched ACC analysis is reported for the 0.5 s, two-calibration condition in Table 10; the longer-window comparisons are descriptive.

At both long windows, the five-subband design produced higher mean ACC and ITR than the single-band design and the no-harmonic reference condition on both datasets. Together with the significant paired results at 0.5 and 1.0 s, this consistent ordering supports the five-subband harmonic reference design used in SQ-HAF.

## 5. Discussion

The experiments examine SQ-HAF as a low-calibration cross-subject SSVEP decoder. Across the 0.5–2.0 s window analysis, longer observation windows increased ACC, whereas ITR peaked at 1.0 s. Recognition performance was lower on BETA than on Benchmark under the evaluated protocol.

### 5.1. Biomedical Relevance and Low-Calibration Operation

The practical motivation for SQ-HAF is to reduce the amount of subject-specific data required before an SSVEP interface can be used. When target calibration is available, the source selection stage uses it to identify previous users with similar stimulus-locked response patterns instead of assigning equal relevance to the entire source pool. In the one-trial condition, the calibration data provide target-specific information for source ranking while maintaining a low calibration burden.

Equation (11) adapts source evaluation to the calibration budget. With one trial per stimulus, g(1)=0 and source ranking is determined by target–source template similarity. With additional calibration trials, STC provides the bounded reliability adjustment defined by the confidence gate.

### 5.2. Roles of Stage 1, Stage 2, and Multi-Branch Fusion

Stage 1 and Stage 2 address different aspects of the transfer problem. Stage 1 learns source-specific spatial filters from repeated trials and their local physical frequency neighborhood. These filters are used in the source-specific template branch. Stage 2 instead aligns subject-level covariance and constructs aligned source templates, from which the generalized template and its cross-subject spatial filter are obtained. Accordingly, Stage 2 covariance is estimated from channel-space trials rather than from the one-dimensional Stage 1 projections.

The controlled five-subband ablation results show that the three decision branches contribute unequally but complementarily. At the one-trial 1.0 s operating point of that component analysis, removing the harmonic reference branch produced the largest branch-specific ACC decrease on both datasets, followed by removal of the generalized source template branch; the source-specific template branch had a smaller aggregate effect. The harmonic reference branch is analytically different from the template branches because it compares the aligned target signal with sinusoidal references across five weighted subbands, whereas the other branches rely on transferred EEG templates. The signed-square fusion also contributed materially: replacing it with absolute value fusion reduced ACC on both datasets.

Stage 2 produced a larger ACC change on BETA than on Benchmark (2.97 versus 0.94 percentage points at the one-trial 1.0 s operating point), indicating a stronger contribution of covariance alignment on BETA under this protocol. Stage 1 had a smaller positive aggregate contribution in the same analysis, with ACC decreasing by 1.64 percentage points on Benchmark and 0.41 percentage points on BETA when it was removed.

### 5.3. Source Reliability, Frequency Neighborhood Regularization, and Harmonic Evidence

The time-ordered STC estimate compares templates formed from early and late trials and therefore reflects both repeatability and slow changes over the recording session. Random and block-stratified partitions produced only small aggregate recognition changes under the tested calibration budgets, and none of the block-stratified versus time-ordered ACC/ITR comparisons remained significant after Holm correction. At $n_{\mathrm{calib}}=1$, the confidence gate is inactive, so the STC partition does not affect the final source quality score. These results characterize STC as a calibration-dependent source-reliability descriptor and show that recognition was stable across the evaluated partition strategies.

Stage 1 regularizes spatial-filter estimation according to physical frequency order. Each neighboring class contributes its own within-class TRCA statistics through the distance-decayed weights in Equation (17), with no cross-class trial pair covariance. The adjacent frequency analysis showed a dataset-dependent effect: Benchmark exhibited fewer adjacent errors with Stage 1 and a Holm-significant paired reduction in the adjacent confusion statistic, whereas BETA showed essentially unchanged aggregate adjacent-error counts and no significant paired difference after correction. The neighborhood strength analysis also produced only small ACC changes across γ=0–1, with no tested value differing significantly from the SQ-HAF setting after Holm correction. This behavior is consistent with local regularization of class-specific spatial statistics rather than temporal or phase averaging across JFPM classes.

The harmonic reference results complement the template-based analysis. In the matched 0.5 s analysis at $n_{\mathrm{calib}}=2$, five-subband processing exceeded single-band processing by 7.4857 percentage points on Benchmark and 7.1321 percentage points on BETA, with both differences remaining significant after Holm correction. The descriptive comparisons at 1.0, 1.5, and 2.0 s showed the same ordering of the three harmonic-processing conditions. At 1.0 s, removing the harmonic reference branch from the complete SQ-HAF configuration also produced the largest branch-specific ACC decrease. Together, these findings support the five-subband harmonic reference design within the fused decision.

### 5.4. Limitations and Future Work

The present evaluation focuses on cross-subject recognition in two public offline SSVEP datasets. Extending the analysis to repeated-session and online BCI use would test the method under gaze transitions, feedback, and longer-term nonstationarity.

Stage 1 uses a fixed local frequency neighborhood, and the main STC definition measures consistency between early and late source templates. The sensitivity analyses showed stable recognition across the tested neighborhood strengths and STC partitions, while the adjacent frequency effect of Stage 1 differed between datasets. Future work can investigate data-adaptive neighborhood construction and reliability measures that distinguish short-term repeatability from slower session variation.

## 6. Conclusions

SQ-HAF combines target-relevant source selection, frequency neighborhood-regularized spatial filtering, inter-subject covariance alignment, and three complementary decision branches for low-calibration cross-subject SSVEP recognition. The harmonic reference branch uses five weighted subbands, while the generalized source template and source-specific template branches transfer complementary information from the selected source subjects.

With one calibration trial per stimulus, the complete SQ-HAF configuration achieved 81.57% ACC and 227.83 bits/min at 1.0 s on Benchmark and 65.56% ACC and 163.42 bits/min on BETA, and it obtained the highest reported ACC and ITR among the compared methods across the 0.5–2.0 s conditions. The 0.5–2.0 s analysis showed that ACC increased with observation length and that mean ITR peaked at 1.0 s. The ablation results showed sizable performance losses when target-relevant source selection was replaced by random selection, when the harmonic reference or generalized source template branch was removed, and when signed-square fusion was replaced by absolute value fusion. In the matched 0.5 s, two-calibration analysis, five-subband harmonic reference processing significantly improved ACC over single-band processing on both datasets after Holm correction. Overall, the results support combining source screening, covariance-aligned template transfer, and complementary harmonic/template evidence for SSVEP recognition with limited target calibration.

## Figures and Tables

**Table 1:** Parameter settings of the compared methods.

Method	Parameter Settings
SQ-HAF	$n_{\mathrm{calib}}=1$; H=3; five harmonic subbands; r=1; γ=1.0; η=0.5; λ=0.2; selected sources $M_{s}=10$ (Benchmark) and 20 (BETA).
SUTL	H=3; number of sub-bands =5; same source subject setting as SQ-HAF.
IISTLF	$n_{\mathrm{calib}}=1$; H=3; one calibration trial per stimulus class; number of sub-bands =5.
EEG-Conformer	supervised convolutional–Transformer architecture; embedding size =40, depth =6, attention heads =10.
eTRCA	H=3; ensemble strategy; number of spatial components =1; passband [5,90] Hz; stopband [3,92] Hz.
TtCCA	H=3; number of canonical components =1.
TDCA	H=3; padding length =1; number of components =4.

**Table 2:** Overall comparison of cross-subject SSVEP recognition performance on the Benchmark and BETA datasets from 0.5 to 2.0 s. Accuracy and ITR are reported as mean ± SD across target subjects. The SQ-HAF entries correspond to the complete configuration with one calibration trial per stimulus; IISTLF also uses one calibration trial per stimulus. The largest reported numerical value under each dataset–window condition is highlighted in bold.

Methods	Metrics	Benchmark Dataset					BETA Dataset			
		0.5 s	1.0 s	1.5 s	2.0 s		0.5 s	1.0 s	1.5 s	2.0 s
SQ-HAF	ACC	**43.81 ± 19.02**	**81.57 ± 17.43**	**90.57 ± 11.57**	**94.96 ± 6.90**		**36.53 ± 18.22**	**65.56 ± 21.10**	**79.34 ± 17.13**	**85.53 ± 14.19**
	ITR	**177.08 ± 110.82**	**227.83 ± 71.66**	**178.81 ± 35.88**	**144.79 ± 18.02**		**135.30 ± 102.62**	**163.42 ± 77.03**	**145.01 ± 47.21**	**122.19 ± 31.19**
EEG-Conformer	ACC	8.46 ± 3.89	35.81 ± 17.63	57.39 ± 22.20	67.14 ± 21.42		7.45 ± 3.90	27.99 ± 14.80	34.84 ± 18.35	49.17 ± 22.92
	ITR	9.48 ± 9.27	65.29 ± 47.53	89.52 ± 47.03	84.76 ± 37.33		7.53 ± 9.05	44.43 ± 36.55	42.26 ± 33.31	53.97 ± 36.15
SUTL	ACC	23.19 ± 11.57	62.13 ± 20.41	78.06 ± 19.03	86.68 ± 14.91		23.23 ± 11.46	50.38 ± 17.60	66.27 ± 17.40	73.97 ± 15.79
	ITR	64.08 ± 52.50	149.60 ± 69.08	141.73 ± 48.68	125.04 ± 31.72		64.03 ± 53.85	107.61 ± 56.55	108.77 ± 43.48	96.51 ± 32.00
IISTLF	ACC	24.25 ± 12.04	65.05 ± 20.08	80.83 ± 18.51	89.20 ± 13.80		18.91 ± 8.91	47.60 ± 19.03	67.04 ± 19.57	76.31 ± 17.47
	ITR	69.04 ± 55.51	160.13 ± 69.66	149.92 ± 49.46	131.27 ± 30.99		44.69 ± 37.06	99.81 ± 59.75	111.84 ± 48.66	102.11 ± 35.24
eTRCA	ACC	31.00 ± 21.29	55.54 ± 27.81	64.40 ± 27.63	73.11 ± 25.11		22.11 ± 12.98	40.50 ± 20.86	51.20 ± 23.04	60.34 ± 24.43
	ITR	113.24 ± 111.26	133.98 ± 93.35	110.67 ± 66.45	99.46 ± 49.28		61.68 ± 58.80	80.62 ± 60.93	76.37 ± 49.29	73.52 ± 41.53
TtCCA	ACC	30.85 ± 17.80	67.44 ± 23.16	80.24 ± 19.57	87.90 ± 13.93		24.24 ± 14.15	49.07 ± 21.99	65.19 ± 22.40	74.04 ± 20.58
	ITR	106.43 ± 93.29	172.55 ± 82.85	149.43 ± 53.71	128.22 ± 31.71		71.50 ± 68.57	106.87 ± 70.78	108.86 ± 54.04	98.66 ± 39.85
TDCA	ACC	28.20 ± 17.44	58.08 ± 24.99	67.70 ± 23.59	76.79 ± 20.90		24.47 ± 13.45	44.46 ± 21.34	55.53 ± 22.58	64.68 ± 23.26
	ITR	93.50 ± 89.98	139.69 ± 85.77	116.21 ± 58.22	105.08 ± 42.92		71.75 ± 64.29	92.33 ± 64.59	85.66 ± 50.19	80.97 ± 41.05

**Table 3:** Effect of target calibration effort on SQ-HAF at 1.0 s. Values are mean ± standard deviation across target subjects.

Dataset	$n_{\mathrm{calib}}$	ACC (%)	ITR (bits/min)
Benchmark	0	74.37 ± 21.18	198.99 ± 81.40
Benchmark	1	81.57 ± 17.43	227.83 ± 71.66
Benchmark	2	81.40 ± 17.60	227.13 ± 71.83
BETA	0	59.79 ± 20.68	141.63 ± 71.69
BETA	1	65.56 ± 21.10	163.42 ± 77.03
BETA	2	65.91 ± 20.85	164.56 ± 76.60

**Table 4:** Controlled ablation results at 1.0 s with one calibration trial per stimulus. Values are mean ± standard deviation across target subjects. All variants share the five-subband SQ-HAF reference configuration except for the component explicitly changed in each row.

Configuration	Benchmark ACC (%)	Benchmark ITR	BETA ACC (%)	BETA ITR
Full five-subband configuration	81.57 ± 17.43	227.83 ± 71.66	65.56 ± 21.10	163.42 ± 77.03
Similarity-only source scoring	81.57 ± 17.43	227.83 ± 71.66	65.56 ± 21.10	163.42 ± 77.03
STC-only source scoring	74.43 ± 20.21	198.38 ± 77.50	60.29 ± 20.11	143.01 ± 69.89
Random source selection	73.29 ± 20.92	194.26 ± 79.48	57.91 ± 20.22	134.67 ± 69.63
Uniform source weighting	81.54 ± 17.46	227.72 ± 71.73	65.54 ± 21.11	163.33 ± 77.04
No Stage 1 spatial filtering	79.93 ± 18.37	221.08 ± 74.39	65.15 ± 21.20	161.89 ± 76.88
No Stage 2 covariance alignment	80.63 ± 18.40	224.36 ± 74.85	62.59 ± 21.91	152.95 ± 78.43
No harmonic reference branch	71.47 ± 20.68	185.97 ± 74.19	50.00 ± 21.98	109.73 ± 70.40
No generalized source template branch	75.59 ± 19.61	202.68 ± 75.58	55.22 ± 20.36	125.51 ± 67.07
No source-specific template branch	80.64 ± 18.16	224.14 ± 73.76	64.94 ± 21.10	161.04 ± 76.60
Absolute-value fusion	69.44 ± 20.16	177.49 ± 72.48	51.10 ± 20.87	112.32 ± 67.18
Random STC partition	81.57 ± 17.43	227.83 ± 71.66	65.56 ± 21.10	163.42 ± 77.03

**Table 5:** Partial adjacent frequency confusion analysis for Stage 1 at 1.0 s and $n_{\mathrm{calib}}=1$. Adjacency is defined by physical stimulation frequency order. Each row is a directed true frequency → predicted frequency cell; Δ is Stage 1 minus no Stage 1.

Dataset	True → Predicted (Hz)	Stage 1	No Stage 1	Δ
Benchmark	12.6 → 12.4	12	23	–11
Benchmark	13.4 → 13.2	13	21	–8
Benchmark	11.0 → 10.8	19	26	–7
Benchmark	13.0 → 12.8	7	13	–6
Benchmark	12.0 → 11.8	19	24	–5
BETA	11.6 → 11.4	30	39	–9
BETA	14.8 → 14.6	52	44	+8
BETA	15.8 → 15.6	44	49	–5
BETA	10.2 → 10.4	9	14	–5
BETA	10.6 → 10.4	32	28	+4

**Table 6:** Paired Stage 1 analysis at 1.0 s and $n_{\mathrm{calib}}=1$. Differences are Stage 1 minus no Stage 1. ACC and adjacent confusion differences are in percentage points (pp), and the reported bootstrap interval is a 95% CI for the mean difference.

Dataset	Metric	*n*	Mean Diff.	Median Diff.	Paired Bootstrap 95% CI	Wilcoxon *p*	$p_{\mathrm{Holm}}$
Benchmark	ACC	35	+1.8524 pp	+1.5000 pp	[+1.2857, +2.4286] pp	$2.897\times 10^{-7}$	$1.159\times 10^{-6}$
Benchmark	Adjacent confusion	35	–1.1830 pp	–1.0380 pp	[–2.2285, –0.1370] pp	0.008854	0.02656
BETA	ACC	70	+0.2103 pp	+0.2778 pp	[–0.0992, +0.5040] pp	0.04915	0.09829
BETA	Adjacent confusion	70	–0.2746 pp	–0.1612 pp	[–0.8000, +0.1949] pp	0.1776	0.1776

**Table 7:** Sensitivity to the Stage 1 neighborhood strength coefficient γ at 1.0 s. Δ ACC is relative to the SQ-HAF setting γ=1.0; $p_{\mathrm{Holm}}$ is the Holm-adjusted paired Wilcoxon *p* value for ACC.

γ	Benchmark Δ ACC (pp)	Benchmark $p_{\mathrm{Holm}}$	BETA Δ ACC (pp)	BETA $p_{\mathrm{Holm}}$
0.0	–0.1191	0.4720	–0.1627	0.0666
0.1	–0.0905	0.9354	–0.0953	0.8353
0.2	–0.0619	1.0000	–0.0516	1.0000
0.4	+0.0095	1.0000	+0.0039	1.0000
0.8	+0.0048	1.0000	+0.0317	0.8353
1.0	0.0000	1.0000	0.0000	1.0000

**Table 8:** Block-stratified versus time-ordered STC partition. Differences are block-stratified minus time-ordered. CIs are paired bootstrap 95% CIs for the mean difference; $p_{\mathrm{Holm}}$ values are Holm-adjusted Wilcoxon signed-rank results.

Dataset	$n_{\mathrm{calib}}$	Metric	Mean Diff.	Paired Bootstrap 95% CI	$p_{\mathrm{Holm}}$
Benchmark	0	ACC (pp)	–0.2143	[–0.4286, –0.0159]	0.3468
Benchmark	0	ITR	–0.9294	[–1.9104, –0.0305]	1.0000
Benchmark	1	ACC (pp)	0.0000	[0.0000, 0.0000]	1.0000
Benchmark	1	ITR	0.0000	[0.0000, 0.0000]	1.0000
Benchmark	2	ACC (pp)	+0.0774	[–0.0476, +0.2202]	1.0000
Benchmark	2	ITR	+0.3678	[–0.1090, +0.9437]	1.0000
BETA	0	ACC (pp)	0.0000	[–0.0208, +0.0208]	0.7330
BETA	0	ITR	–0.0273	[–0.0971, +0.0357]	0.9322
BETA	1	ACC (pp)	0.0000	[0.0000, 0.0000]	1.0000
BETA	1	ITR	0.0000	[0.0000, 0.0000]	1.0000
BETA	2	ACC (pp)	+0.0238	[–0.1071, +0.1607]	1.0000
BETA	2	ITR	+0.0809	[–0.3642, +0.5990]	1.0000

**Table 9:** Harmonic reference comparison at 0.5 s. Each cell reports mean ACC (%)/mean ITR (bits/min).

Dataset	Harmonic Reference Design	$n_{\mathrm{calib}}=0$	$n_{\mathrm{calib}}=1$	$n_{\mathrm{calib}}=2$
Benchmark	Five-subband	38.64/147.97	39.14/149.93	39.28/150.94
Benchmark	Single-band	30.94/104.69	31.71/108.12	31.79/108.60
Benchmark	No harmonic reference branch	29.52/96.56	30.13/97.98	30.22/98.62
BETA	Five-subband	33.66/118.00	35.10/126.18	35.35/127.63
BETA	Single-band	26.74/83.34	28.02/90.40	28.21/91.62
BETA	No harmonic reference branch	23.12/65.34	24.84/73.54	24.91/74.33

**Table 10:** Matched 0.5 s ACC comparisons at $n_{\mathrm{calib}}=2$. Differences are expressed in percentage points (pp). The 95% CI is the paired *t*-based interval for the mean difference at the target–seed level; $p_{\mathrm{Holm}}$ denotes the Holm-adjusted Wilcoxon signed-rank *p* value. The Holm family also included the prespecified Stage 1, STC-partition, and source confidence comparisons from the same 0.5 s analysis.

Dataset	Comparison	Mean Diff.	95% CI	Wilcoxon *p*	$p_{\mathrm{Holm}}$
Benchmark	Five-subband − single-band	+7.4857 pp	[6.6176, 8.3539] pp	$6.263\times 10^{-28}$	$8.769\times 10^{-27}$
Benchmark	Single-band − no harmonic reference	+1.5679 pp	[0.2929, 2.8428] pp	0.3249	1.0000
BETA	Five-subband − single-band	+7.1321 pp	[6.5339, 7.7304] pp	$6.572\times 10^{-51}$	$9.201\times 10^{-50}$
BETA	Single-band − no harmonic reference	+3.3071 pp	[2.5855, 4.0288] pp	$2.257\times 10^{-15}$	$2.708\times 10^{-14}$

**Table 11:** Harmonic reference comparison at 1.5 and 2.0 s with one calibration trial per stimulus. Each cell reports mean ACC (%)/mean ITR (bits/min).

Dataset	Harmonic Reference Design	1.5 s	2.0 s
Benchmark	Five-subband	89.11/174.49	94.03/142.34
Benchmark	Single-band	79.11/146.05	86.44/124.84
Benchmark	No harmonic reference branch	74.38/132.10	83.45/117.84
BETA	Five-subband	78.62/142.87	84.90/120.61
BETA	Single-band	65.30/109.95	73.35/97.80
BETA	No harmonic reference branch	56.21/87.33	64.77/80.98

## Data Availability

The Benchmark Dataset for SSVEP-Based Brain–Computer Interfaces [8] and the BETA database [9] are publicly available from the Brain–Computer Interface Research Group, Tsinghua University, at https://bci.med.tsinghua.edu.cn/ (accessed on 8 September 2026). Their original descriptions are identified by DOIs https://doi.org/10.1109/TNSRE.2016.2627556 (accessed on 8 September 2026) and https://doi.org/10.3389/fnins.2020.00627 (accessed on 8 September 2026), respectively. The source code used in this study is publicly available at https://github.com/foamzzz/SQHAF (accessed on 8 September 2026).

## Appendix A. Additional 1.0 s Structural Ablation Results

Table A1 and Table A2 report the ACC summaries for the principal structural ablations across zero, one, and two calibration trials per stimulus. The one-trial ACC/ITR results for the broader ablation set are reported in Table 4; no additional interpretation is repeated here.

**Table A1:** Structural ablation ACC results on Benchmark at 1.0 s. Values are mean ± standard deviation (%).

Configuration	$n_{\mathrm{calib}}=0$	$n_{\mathrm{calib}}=1$	$n_{\mathrm{calib}}=2$
Full five-subband configuration	74.3690 ± 21.1803	81.5686 ± 17.4349	81.4000 ± 17.5966
No harmonic reference branch	56.7500 ± 24.9450	71.4657 ± 20.6824	70.6821 ± 20.6307
No Stage 2 covariance alignment	72.6310 ± 21.8204	80.6286 ± 18.4006	80.1571 ± 18.8598
No Stage 1 spatial filtering	74.0833 ± 20.9484	79.9257 ± 18.3736	79.7786 ± 18.3265
No generalized source template branch	70.4405 ± 21.7971	75.5943 ± 19.6086	75.5536 ± 19.4634

**Table A2:** Structural ablation ACC results on BETA at 1.0 s. Values are mean ± standard deviation (%).

Configuration	$n_{\mathrm{calib}}=0$	$n_{\mathrm{calib}}=1$	$n_{\mathrm{calib}}=2$
Full five-subband configuration	59.7946 ± 20.6840	65.5619 ± 21.1040	65.9107 ± 20.8495
No harmonic reference branch	38.6518 ± 21.9634	50.0000 ± 21.9796	51.0893 ± 21.6751
No Stage 2 covariance alignment	58.6071 ± 21.0759	62.5881 ± 21.9109	62.5357 ± 22.0994
No Stage 1 spatial filtering	59.1071 ± 20.5659	65.1476 ± 21.1967	65.3464 ± 21.0192
No generalized source template branch	50.6875 ± 19.4102	55.2238 ± 20.3610	56.3536 ± 20.3797
